# {*N*-[(2-Oxido-1-naphth­yl)methyl­idene]­serinato-κ^3^ *O*,*N*,*O*′}(1,10-phenanthroline-κ^2^ *N*,*N*′)copper(II)

**DOI:** 10.1107/S1600536810012675

**Published:** 2010-04-10

**Authors:** Jinghong Li, Zhenghua Guo, Lianzhi Li, Daqi Wang

**Affiliations:** a School of Chemistry and Chemical Engineering, Liaocheng University, Shandong 252059, People’s Republic of China

## Abstract

In the title complex, [Cu(C_14_H_11_NO_4_)(C_12_H_8_N_2_)], the tridentate Schiff base ligand is derived from the condensation of 2-hydr­oxy-1-naphthaldehyde and l-serine. The Cu^II^ atom is five-coordinated by one N atom and two O atoms from the Schiff base ligand and by two N atoms from a 1,10-phenanthroline ligand in a distorted square-pyramidal geometry. In the crystal structure, the combination of inter­molecular O—H⋯O and C—H⋯O hydrogen bonds results in a two-dimensional network structure parallel to (001).

## Related literature

For general background to Schiff base complexes, see: Garnovski *et al.* (1993 ▶); Kalagouda *et al.* (2006 ▶); Wang *et al.* (1999 ▶). For our previous work on amino Schiff base complexes, see: Qiu *et al.* (2008 ▶); Wang *et al.* (2007 ▶).
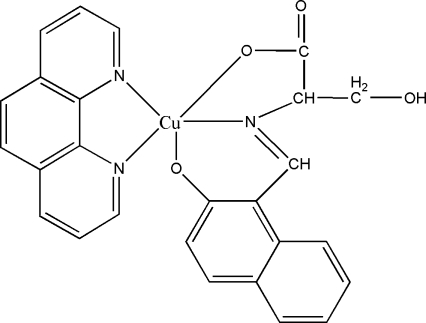


## Experimental

### 

#### Crystal data

[Cu(C_14_H_11_NO_4_)(C_12_H_8_N_2_)]
*M* *_r_* = 500.98
Monoclinic, 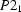

*a* = 10.7302 (12) Å
*b* = 6.4687 (6) Å
*c* = 15.7930 (17) Å
β = 91.924 (1)°
*V* = 1095.6 (2) Å^3^
*Z* = 2
Mo *K*α radiation
μ = 1.04 mm^−1^
*T* = 298 K
0.43 × 0.16 × 0.08 mm

#### Data collection

Bruker SMART 1000 CCD area-detector diffractometer
Absorption correction: multi-scan (*SADABS*; Sheldrick, 1996 ▶) *T*_min_ = 0.664, *T*_max_ = 0.922
5555 measured reflections
3633 independent reflections
3022 reflections with *I* > 2σ(*I*)
*R*_int_ = 0.031

#### Refinement

*R*[*F* ^2^ > 2σ(*F* ^2^)] = 0.041
*wR*(*F* ^2^) = 0.094
*S* = 0.97
3633 reflections
307 parameters
1 restraint
H-atom parameters constrained
Δρ_max_ = 0.41 e Å^−3^
Δρ_min_ = −0.25 e Å^−3^
Absolute structure: Flack (1983 ▶), with 1529 Friedel pairs
Flack parameter: −0.023 (17)

### 

Data collection: *SMART* (Bruker, 2007 ▶); cell refinement: *SAINT* (Bruker, 2007 ▶); data reduction: *SAINT*; program(s) used to solve structure: *SHELXS97* (Sheldrick, 2008 ▶); program(s) used to refine structure: *SHELXL97* (Sheldrick, 2008 ▶); molecular graphics: *SHELXTL* (Sheldrick, 2008 ▶) and *DIAMOND* (Brandenburg, 1999 ▶); software used to prepare material for publication: *SHELXTL*.

## Supplementary Material

Crystal structure: contains datablocks global, I. DOI: 10.1107/S1600536810012675/hy2297sup1.cif

Structure factors: contains datablocks I. DOI: 10.1107/S1600536810012675/hy2297Isup2.hkl

Additional supplementary materials:  crystallographic information; 3D view; checkCIF report

## Figures and Tables

**Table 1:** Selected bond lengths (Å)

Cu1—N1	1.914 (3)
Cu1—N2	2.012 (4)
Cu1—N3	2.297 (4)
Cu1—O1	1.994 (3)
Cu1—O4	1.920 (3)

**Table 2:** Hydrogen-bond geometry (Å, °)

*D*—H⋯*A*	*D*—H	H⋯*A*	*D*⋯*A*	*D*—H⋯*A*
O3—H3⋯O2^i^	0.82	1.84	2.659 (5)	172
C25—H25⋯O2^ii^	0.93	2.63	3.454 (6)	148

Symmetry codes: (i) 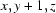; (ii) 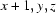.

**Fig. 1.**
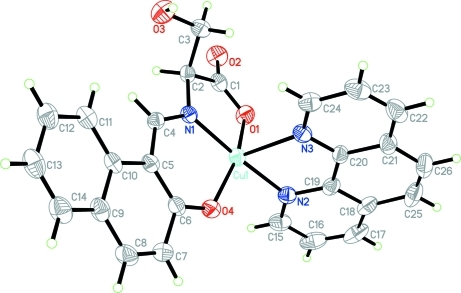
The molecular structure of the title compound, showing 30% probability displacement ellipsoids.

**Fig. 2.**
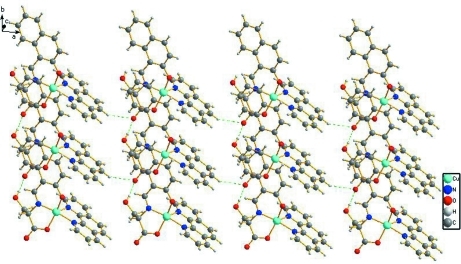
Packing diagram of the title compound with hydrogen bonds shown as dashed lines.

## supplementary crystallographic information

### Comment

Amino acids are very important biomolecules because of their roles in biochemical reactions. Schiff base complexes have continued to play the role of the most important stereochemical models in main group and transition metal coordination chemistry with their easy preparation and structural variation (Garnovski *et al.*, 1993). So efforts have been made to synthesize and characterize amino Schiff base complexes with transition metals, and more and more these new complexes have been reported (Kalagouda *et al.*, 2006; Wang *et al.*, 1999). As part of a series of our study (Qiu *et al.* 2008; Wang *et al.*, 2007), we report here the synthesis and crystal structure of a new copper(II) complex with a tridentate Schiff base ligand derived from the condensation of 2-hydroxy-1-naphthaldehyde and L-serine.

The molecular structure of the title complex is shown in Fig. 1. The Cu^II^ atom is five-coordinated with two O atom and one N atom from a tridentate Schiff base ligand, and two N atoms from a 1,10-phenanthroline ligand, resulting in a distorted square-pyramidal geometry. O1, O4, N1 and N2 locate in a basal equatorial plane and N3 is at the apical position. The Cu^II^ atom deviates from the basal equatorial plane by 0.2005 (18) Å toward N3 atom, with a significantly longer Cu1—N3 bond distance [2.297 (4) Å] (Table 1). The apical Cu1—N3 bond deviates greatly from the right position to close the Cu1—N2 bond [N2—Cu1—N3 = 77.87 (14)°]. Additionally, the tridentate Schiff base ligand coordinates to the Cu atom, forming two chelating rings (Cu1, O1, C1, C2, N1 ring and Cu1, N1, C4, C5, C6, O4 ring). The two rings have dihedral angles of 10.84 (21) and 6.74 (21)° to the equatorial plane, respectively. The 1,10-phenanthroline chelating ring (Cu1, N2, C19, C20, N3) is almost perpendicular to the basal equatorial plane [dihedral angle = 85.91 (9)°]. In the crystal, the combination of intermolecular O—H···O and C—H···O hydrogen bonds (Table 2) leads to a two-dimensional network (Fig. 2).

### Experimental

L-Serine (1 mmol, 105.1 mg) and potassium hydroxide (1 mmol, 56.1 mg) were dissovlved in hot methanol (5 ml) and added in portions to a methanol solution of 2-hydroxy-1-naphthaldehyde (1 mmol, 172.19 mg). The mixture was then stirred at 323 K for 2 h. Subsequently, an aqueous solution (2 ml) of cupric acetate monohydrate (1 mmol, 199.7 mg) was added dropwise and the mixture stirred for 3 h. Finally, a methanol solution (5 ml) of 1,10-phenanthroline (1 mmol, 198.2 mg) was added dropwise to the above solution and then stirred for 3 h. The solution was held at room temperature for 15 d, whereupon green needle crystals suitable for X-ray diffraction were obtained.

### Refinement

H atoms were positioned geometrically and refined as riding atoms, with C—H = 0.93–0.98 Å and O—H = 0.82 Å, and with *U*_iso_(H) = 1.2*U*_eq_(C) or 1.5*U*_eq_(O) for hydroxyl group.

### Crystal data

**Table:**

[Cu(C_14_H_11_NO_4_)(C_12_H_8_N_2_)]	*F*(000) = 514
*M**_r_* = 500.98	*D*_x_ = 1.519 Mg m^−^^3^
Monoclinic, *P*2_1_	Mo *K*α radiation, λ = 0.71073 Å
Hall symbol: P 2yb	Cell parameters from 746 reflections
*a* = 10.7302 (12) Å	θ = 3.3–25.2°
*b* = 6.4687 (6) Å	µ = 1.04 mm^−^^1^
*c* = 15.7930 (17) Å	*T* = 298 K
β = 91.924 (1)°	Needle, green
*V* = 1095.6 (2) Å^3^	0.43 × 0.16 × 0.08 mm
*Z* = 2	

### Data collection

**Table:**

Bruker SMART 1000 CCD area-detector diffractometer	3633 independent reflections
Radiation source: fine-focus sealed tube	3022 reflections with *I* > 2σ(*I*)
graphite	*R*_int_ = 0.031
φ and ω scans	θ_max_ = 25.0°, θ_min_ = 1.9°
Absorption correction: multi-scan (*SADABS*; Sheldrick, 1996)	*h* = −12→11
*T*_min_ = 0.664, *T*_max_ = 0.922	*k* = −7→7
5555 measured reflections	*l* = −18→15

### Refinement

**Table:**

Refinement on *F*^2^	Secondary atom site location: difference Fourier map
Least-squares matrix: full	Hydrogen site location: inferred from neighbouring sites
*R*[*F*^2^ > 2σ(*F*^2^)] = 0.041	H-atom parameters constrained
*wR*(*F*^2^) = 0.094	*w* = 1/[σ^2^(*F*_o_^2^) + (0.0491*P*)^2^] where *P* = (*F*_o_^2^ + 2*F*_c_^2^)/3
*S* = 0.97	(Δ/σ)_max_ = 0.001
3633 reflections	Δρ_max_ = 0.41 e Å^−^^3^
307 parameters	Δρ_min_ = −0.25 e Å^−^^3^
1 restraint	Absolute structure: Flack (1983), with 1529 Friedel pairs
Primary atom site location: structure-invariant direct methods	Flack parameter: −0.023 (17)

### Fractional atomic coordinates and isotropic or equivalent isotropic displacement parameters (Å^2^)

**Table:**

	*x*	*y*	*z*	*U*_iso_*/*U*_eq_	
Cu1	0.85224 (4)	0.14981 (9)	0.75536 (3)	0.04385 (16)	
N1	0.6939 (3)	0.2834 (6)	0.7406 (2)	0.0385 (8)	
N2	1.0094 (3)	−0.0200 (6)	0.7612 (2)	0.0472 (9)	
N3	0.9778 (3)	0.3251 (6)	0.6648 (2)	0.0455 (9)	
O1	0.7806 (3)	−0.0536 (5)	0.6722 (2)	0.0536 (8)	
O2	0.6057 (4)	−0.1294 (5)	0.5986 (2)	0.0629 (10)	
O3	0.5115 (3)	0.4941 (6)	0.6180 (2)	0.0682 (10)	
H3	0.5463	0.6051	0.6103	0.102*	
O4	0.8869 (3)	0.2791 (5)	0.86299 (18)	0.0534 (8)	
C1	0.6697 (4)	−0.0168 (7)	0.6467 (3)	0.0455 (11)	
C2	0.6124 (3)	0.1876 (7)	0.6743 (2)	0.0394 (11)	
H2	0.5311	0.1589	0.6983	0.047*	
C3	0.5927 (5)	0.3310 (8)	0.5981 (3)	0.0497 (12)	
H3A	0.6723	0.3871	0.5820	0.060*	
H3B	0.5578	0.2532	0.5505	0.060*	
C4	0.6521 (4)	0.4288 (7)	0.7864 (3)	0.0406 (10)	
H4	0.5715	0.4751	0.7738	0.049*	
C5	0.7188 (4)	0.5272 (7)	0.8559 (3)	0.0412 (10)	
C6	0.8261 (4)	0.4355 (8)	0.8927 (3)	0.0463 (11)	
C7	0.8738 (4)	0.5219 (9)	0.9718 (3)	0.0568 (13)	
H7	0.9410	0.4577	0.9997	0.068*	
C8	0.8240 (4)	0.6931 (9)	1.0064 (3)	0.0602 (15)	
H8	0.8578	0.7431	1.0574	0.072*	
C9	0.7211 (4)	0.7988 (8)	0.9669 (3)	0.0524 (12)	
C10	0.6680 (4)	0.7142 (6)	0.8910 (3)	0.0434 (12)	
C11	0.5671 (4)	0.8226 (8)	0.8529 (3)	0.0534 (12)	
H11	0.5300	0.7711	0.8032	0.064*	
C12	0.5216 (5)	1.0034 (8)	0.8871 (3)	0.0596 (13)	
H12	0.4540	1.0702	0.8607	0.071*	
C13	0.5764 (5)	1.0859 (8)	0.9608 (3)	0.0642 (15)	
H13	0.5467	1.2085	0.9833	0.077*	
C14	0.6735 (5)	0.9856 (9)	0.9992 (3)	0.0636 (14)	
H14	0.7099	1.0412	1.0484	0.076*	
C15	1.0230 (5)	−0.1874 (8)	0.8073 (3)	0.0621 (14)	
H15	0.9624	−0.2191	0.8460	0.075*	
C16	1.1243 (5)	−0.3193 (11)	0.8008 (4)	0.0776 (17)	
H16	1.1298	−0.4381	0.8339	0.093*	
C17	1.2155 (5)	−0.2738 (9)	0.7458 (4)	0.0780 (18)	
H17	1.2840	−0.3608	0.7415	0.094*	
C18	1.2056 (4)	−0.0959 (8)	0.6958 (3)	0.0564 (13)	
C19	1.0996 (4)	0.0292 (8)	0.7055 (3)	0.0481 (11)	
C20	1.0833 (4)	0.2127 (7)	0.6554 (3)	0.0429 (12)	
C21	1.1751 (4)	0.2679 (9)	0.5972 (3)	0.0548 (12)	
C22	1.1553 (5)	0.4525 (10)	0.5521 (3)	0.0694 (16)	
H22	1.2140	0.4970	0.5141	0.083*	
C23	1.0512 (5)	0.5663 (8)	0.5637 (3)	0.0671 (15)	
H23	1.0383	0.6891	0.5340	0.080*	
C24	0.9638 (4)	0.4976 (8)	0.6204 (3)	0.0552 (12)	
H24	0.8924	0.5764	0.6274	0.066*	
C25	1.2954 (4)	−0.0334 (11)	0.6350 (4)	0.0730 (17)	
H25	1.3658	−0.1147	0.6282	0.088*	
C26	1.2811 (4)	0.1357 (14)	0.5886 (3)	0.0708 (15)	
H26	1.3409	0.1694	0.5496	0.085*	

### Atomic displacement parameters (Å^2^)

**Table:**

	*U*^11^	*U*^22^	*U*^33^	*U*^12^	*U*^13^	*U*^23^
Cu1	0.0443 (3)	0.0443 (3)	0.0434 (3)	0.0024 (3)	0.00652 (18)	−0.0022 (3)
N1	0.0436 (18)	0.036 (2)	0.0364 (18)	−0.0015 (16)	0.0031 (15)	−0.0063 (16)
N2	0.050 (2)	0.046 (2)	0.046 (2)	−0.0002 (18)	−0.0006 (17)	0.0024 (19)
N3	0.052 (2)	0.042 (2)	0.043 (2)	−0.0019 (19)	0.0020 (16)	0.0027 (18)
O1	0.0538 (19)	0.048 (2)	0.059 (2)	0.0080 (16)	0.0002 (16)	−0.0130 (17)
O2	0.075 (2)	0.048 (2)	0.066 (2)	−0.002 (2)	−0.0035 (19)	−0.0200 (19)
O3	0.068 (2)	0.047 (2)	0.089 (3)	0.0030 (19)	−0.0033 (19)	0.002 (2)
O4	0.0529 (17)	0.063 (2)	0.0442 (17)	0.0077 (17)	−0.0007 (14)	−0.0051 (16)
C1	0.054 (3)	0.040 (3)	0.043 (2)	−0.006 (2)	0.008 (2)	−0.006 (2)
C2	0.0393 (19)	0.040 (3)	0.039 (2)	−0.005 (2)	0.0038 (16)	−0.011 (2)
C3	0.058 (3)	0.046 (3)	0.045 (3)	−0.006 (3)	0.000 (2)	0.002 (2)
C4	0.039 (2)	0.041 (3)	0.042 (2)	−0.002 (2)	0.0012 (18)	−0.002 (2)
C5	0.046 (2)	0.042 (3)	0.036 (2)	−0.006 (2)	0.0052 (19)	−0.004 (2)
C6	0.047 (2)	0.053 (3)	0.039 (2)	−0.007 (2)	0.0077 (19)	−0.004 (2)
C7	0.051 (3)	0.072 (4)	0.047 (3)	−0.001 (3)	−0.003 (2)	−0.006 (3)
C8	0.066 (3)	0.069 (5)	0.045 (2)	−0.011 (3)	−0.002 (2)	−0.022 (3)
C9	0.057 (3)	0.050 (3)	0.051 (3)	−0.010 (2)	0.012 (2)	−0.014 (2)
C10	0.054 (2)	0.042 (3)	0.036 (2)	−0.007 (2)	0.0115 (19)	−0.0030 (18)
C11	0.064 (3)	0.052 (3)	0.045 (3)	−0.004 (3)	0.011 (2)	−0.003 (2)
C12	0.073 (3)	0.048 (3)	0.058 (3)	0.004 (3)	0.018 (3)	−0.003 (3)
C13	0.085 (4)	0.047 (4)	0.061 (3)	−0.002 (3)	0.024 (3)	−0.013 (2)
C14	0.075 (3)	0.058 (4)	0.058 (3)	−0.013 (3)	0.010 (3)	−0.024 (3)
C15	0.072 (3)	0.054 (3)	0.060 (3)	−0.003 (3)	−0.012 (3)	0.011 (3)
C16	0.087 (3)	0.054 (4)	0.090 (4)	0.010 (4)	−0.035 (3)	0.014 (4)
C17	0.057 (3)	0.068 (4)	0.106 (5)	0.015 (3)	−0.031 (3)	−0.014 (3)
C18	0.041 (3)	0.055 (3)	0.071 (3)	0.006 (2)	−0.012 (2)	−0.017 (3)
C19	0.039 (2)	0.052 (3)	0.053 (3)	−0.001 (2)	−0.005 (2)	−0.014 (2)
C20	0.037 (2)	0.049 (3)	0.043 (2)	−0.0048 (19)	0.0044 (18)	−0.006 (2)
C21	0.048 (3)	0.065 (3)	0.052 (3)	−0.014 (3)	0.008 (2)	−0.010 (3)
C22	0.076 (4)	0.080 (4)	0.053 (3)	−0.033 (3)	0.008 (3)	0.000 (3)
C23	0.087 (4)	0.057 (4)	0.057 (3)	−0.019 (3)	−0.008 (3)	0.013 (2)
C24	0.060 (3)	0.047 (3)	0.058 (3)	−0.004 (2)	−0.010 (2)	0.004 (2)
C25	0.041 (3)	0.082 (5)	0.097 (5)	0.003 (3)	0.009 (3)	−0.035 (4)
C26	0.049 (3)	0.092 (4)	0.072 (3)	−0.013 (4)	0.018 (2)	−0.027 (5)

### Geometric parameters (Å, °)

**Table:**

Cu1—N1	1.914 (3)	C9—C14	1.415 (7)
Cu1—N2	2.012 (4)	C9—C10	1.419 (6)
Cu1—N3	2.297 (4)	C10—C11	1.407 (6)
Cu1—O1	1.994 (3)	C11—C12	1.385 (7)
Cu1—O4	1.920 (3)	C11—H11	0.9300
N1—C4	1.278 (5)	C12—C13	1.393 (7)
N1—C2	1.477 (5)	C12—H12	0.9300
N2—C15	1.310 (6)	C13—C14	1.353 (7)
N2—C19	1.367 (6)	C13—H13	0.9300
N3—C24	1.324 (6)	C14—H14	0.9300
N3—C20	1.358 (5)	C15—C16	1.388 (7)
O1—C1	1.267 (5)	C15—H15	0.9300
O2—C1	1.242 (5)	C16—C17	1.363 (8)
O3—C3	1.411 (6)	C16—H16	0.9300
O3—H3	0.8200	C17—C18	1.398 (8)
O4—C6	1.300 (5)	C17—H17	0.9300
C1—C2	1.527 (6)	C18—C19	1.408 (6)
C2—C3	1.528 (6)	C18—C25	1.441 (8)
C2—H2	0.9800	C19—C20	1.434 (6)
C3—H3A	0.9700	C20—C21	1.415 (6)
C3—H3B	0.9700	C21—C22	1.403 (8)
C4—C5	1.438 (6)	C21—C26	1.433 (8)
C4—H4	0.9300	C22—C23	1.356 (8)
C5—C6	1.404 (6)	C22—H22	0.9300
C5—C10	1.445 (6)	C23—C24	1.391 (7)
C6—C7	1.447 (6)	C23—H23	0.9300
C7—C8	1.353 (7)	C24—H24	0.9300
C7—H7	0.9300	C25—C26	1.323 (9)
C8—C9	1.424 (7)	C25—H25	0.9300
C8—H8	0.9300	C26—H26	0.9300
			
N1—Cu1—O4	93.23 (13)	C14—C9—C8	122.4 (5)
N1—Cu1—O1	84.06 (14)	C10—C9—C8	117.9 (4)
O4—Cu1—O1	158.33 (14)	C11—C10—C9	116.8 (4)
N1—Cu1—N2	172.54 (16)	C11—C10—C5	123.2 (4)
O4—Cu1—N2	93.43 (14)	C9—C10—C5	120.0 (4)
O1—Cu1—N2	88.51 (14)	C12—C11—C10	122.0 (4)
N1—Cu1—N3	103.78 (14)	C12—C11—H11	119.0
O4—Cu1—N3	103.66 (14)	C10—C11—H11	119.0
O1—Cu1—N3	97.86 (13)	C11—C12—C13	120.3 (5)
N2—Cu1—N3	77.87 (14)	C11—C12—H12	119.8
C4—N1—C2	120.0 (3)	C13—C12—H12	119.8
C4—N1—Cu1	126.2 (3)	C14—C13—C12	119.2 (5)
C2—N1—Cu1	113.5 (3)	C14—C13—H13	120.4
C15—N2—C19	118.8 (4)	C12—C13—H13	120.4
C15—N2—Cu1	123.7 (3)	C13—C14—C9	122.0 (5)
C19—N2—Cu1	117.0 (3)	C13—C14—H14	119.0
C24—N3—C20	118.2 (4)	C9—C14—H14	119.0
C24—N3—Cu1	133.4 (3)	N2—C15—C16	122.7 (5)
C20—N3—Cu1	108.2 (3)	N2—C15—H15	118.7
C1—O1—Cu1	115.0 (3)	C16—C15—H15	118.7
C3—O3—H3	109.5	C17—C16—C15	119.7 (6)
C6—O4—Cu1	125.0 (3)	C17—C16—H16	120.2
O2—C1—O1	125.3 (4)	C15—C16—H16	120.2
O2—C1—C2	117.5 (4)	C16—C17—C18	119.7 (5)
O1—C1—C2	117.1 (4)	C16—C17—H17	120.2
N1—C2—C1	109.3 (3)	C18—C17—H17	120.2
N1—C2—C3	111.4 (4)	C17—C18—C19	117.3 (5)
C1—C2—C3	110.3 (3)	C17—C18—C25	124.6 (5)
N1—C2—H2	108.6	C19—C18—C25	118.1 (5)
C1—C2—H2	108.6	N2—C19—C18	121.8 (5)
C3—C2—H2	108.6	N2—C19—C20	118.2 (4)
O3—C3—C2	110.4 (4)	C18—C19—C20	120.0 (4)
O3—C3—H3A	109.6	N3—C20—C21	122.5 (4)
C2—C3—H3A	109.6	N3—C20—C19	118.0 (4)
O3—C3—H3B	109.6	C21—C20—C19	119.5 (4)
C2—C3—H3B	109.6	C22—C21—C20	116.6 (5)
H3A—C3—H3B	108.1	C22—C21—C26	124.5 (5)
N1—C4—C5	125.7 (4)	C20—C21—C26	118.9 (5)
N1—C4—H4	117.2	C23—C22—C21	120.3 (5)
C5—C4—H4	117.2	C23—C22—H22	119.8
C6—C5—C4	120.6 (4)	C21—C22—H22	119.8
C6—C5—C10	120.6 (4)	C22—C23—C24	119.3 (5)
C4—C5—C10	118.6 (4)	C22—C23—H23	120.3
O4—C6—C5	126.5 (4)	C24—C23—H23	120.3
O4—C6—C7	116.4 (4)	N3—C24—C23	122.9 (5)
C5—C6—C7	117.1 (4)	N3—C24—H24	118.5
C8—C7—C6	122.1 (5)	C23—C24—H24	118.5
C8—C7—H7	118.9	C26—C25—C18	122.3 (5)
C6—C7—H7	118.9	C26—C25—H25	118.9
C7—C8—C9	121.8 (4)	C18—C25—H25	118.9
C7—C8—H8	119.1	C25—C26—C21	121.2 (5)
C9—C8—H8	119.1	C25—C26—H26	119.4
C14—C9—C10	119.6 (5)	C21—C26—H26	119.4
			
O4—Cu1—N1—C4	9.5 (4)	C7—C8—C9—C10	2.6 (7)
O1—Cu1—N1—C4	168.0 (4)	C14—C9—C10—C11	−1.2 (6)
N3—Cu1—N1—C4	−95.4 (4)	C8—C9—C10—C11	−179.5 (4)
O4—Cu1—N1—C2	−164.0 (3)	C14—C9—C10—C5	177.9 (4)
O1—Cu1—N1—C2	−5.6 (3)	C8—C9—C10—C5	−0.4 (6)
N3—Cu1—N1—C2	91.1 (3)	C6—C5—C10—C11	174.5 (4)
O4—Cu1—N2—C15	77.5 (4)	C4—C5—C10—C11	−10.0 (6)
O1—Cu1—N2—C15	−80.9 (4)	C6—C5—C10—C9	−4.5 (6)
N3—Cu1—N2—C15	−179.3 (4)	C4—C5—C10—C9	170.9 (4)
O4—Cu1—N2—C19	−111.2 (3)	C9—C10—C11—C12	0.2 (6)
O1—Cu1—N2—C19	90.4 (3)	C5—C10—C11—C12	−178.9 (4)
N3—Cu1—N2—C19	−7.9 (3)	C10—C11—C12—C13	0.9 (7)
N1—Cu1—N3—C24	10.5 (4)	C11—C12—C13—C14	−1.0 (7)
O4—Cu1—N3—C24	−86.3 (4)	C12—C13—C14—C9	0.0 (7)
O1—Cu1—N3—C24	96.3 (4)	C10—C9—C14—C13	1.2 (7)
N2—Cu1—N3—C24	−176.9 (4)	C8—C9—C14—C13	179.4 (5)
N1—Cu1—N3—C20	−165.7 (3)	C19—N2—C15—C16	−1.2 (7)
O4—Cu1—N3—C20	97.4 (3)	Cu1—N2—C15—C16	170.0 (4)
O1—Cu1—N3—C20	−80.0 (3)	N2—C15—C16—C17	1.2 (8)
N2—Cu1—N3—C20	6.8 (3)	C15—C16—C17—C18	−0.6 (8)
N1—Cu1—O1—C1	−0.8 (3)	C16—C17—C18—C19	0.1 (8)
O4—Cu1—O1—C1	83.0 (5)	C16—C17—C18—C25	−179.4 (5)
N2—Cu1—O1—C1	178.6 (3)	C15—N2—C19—C18	0.6 (7)
N3—Cu1—O1—C1	−103.9 (3)	Cu1—N2—C19—C18	−171.2 (3)
N1—Cu1—O4—C6	−13.8 (4)	C15—N2—C19—C20	179.9 (4)
O1—Cu1—O4—C6	−95.8 (5)	Cu1—N2—C19—C20	8.1 (5)
N2—Cu1—O4—C6	169.6 (3)	C17—C18—C19—N2	0.0 (7)
N3—Cu1—O4—C6	91.3 (3)	C25—C18—C19—N2	179.4 (4)
Cu1—O1—C1—O2	−176.0 (4)	C17—C18—C19—C20	−179.3 (4)
Cu1—O1—C1—C2	6.9 (5)	C25—C18—C19—C20	0.1 (6)
C4—N1—C2—C1	−164.0 (4)	C24—N3—C20—C21	−3.1 (6)
Cu1—N1—C2—C1	10.0 (4)	Cu1—N3—C20—C21	173.8 (4)
C4—N1—C2—C3	73.8 (5)	C24—N3—C20—C19	178.2 (4)
Cu1—N1—C2—C3	−112.2 (3)	Cu1—N3—C20—C19	−4.9 (4)
O2—C1—C2—N1	171.6 (4)	N2—C19—C20—N3	−1.4 (6)
O1—C1—C2—N1	−11.1 (5)	C18—C19—C20—N3	177.9 (4)
O2—C1—C2—C3	−65.5 (5)	N2—C19—C20—C21	179.9 (4)
O1—C1—C2—C3	111.8 (4)	C18—C19—C20—C21	−0.8 (6)
N1—C2—C3—O3	−73.8 (4)	N3—C20—C21—C22	3.2 (7)
C1—C2—C3—O3	164.6 (4)	C19—C20—C21—C22	−178.1 (4)
C2—N1—C4—C5	176.3 (4)	N3—C20—C21—C26	−177.3 (4)
Cu1—N1—C4—C5	3.1 (6)	C19—C20—C21—C26	1.4 (7)
N1—C4—C5—C6	−16.1 (7)	C20—C21—C22—C23	−1.4 (8)
N1—C4—C5—C10	168.5 (4)	C26—C21—C22—C23	179.1 (5)
Cu1—O4—C6—C5	5.7 (6)	C21—C22—C23—C24	−0.4 (8)
Cu1—O4—C6—C7	−174.7 (3)	C20—N3—C24—C23	1.1 (7)
C4—C5—C6—O4	11.2 (7)	Cu1—N3—C24—C23	−174.8 (3)
C10—C5—C6—O4	−173.4 (4)	C22—C23—C24—N3	0.6 (8)
C4—C5—C6—C7	−168.4 (4)	C17—C18—C25—C26	179.3 (5)
C10—C5—C6—C7	7.0 (6)	C19—C18—C25—C26	−0.1 (8)
O4—C6—C7—C8	175.5 (4)	C18—C25—C26—C21	0.7 (9)
C5—C6—C7—C8	−4.8 (7)	C22—C21—C26—C25	178.1 (5)
C6—C7—C8—C9	0.0 (7)	C20—C21—C26—C25	−1.4 (8)
C7—C8—C9—C14	−175.6 (5)		

### Hydrogen-bond geometry (Å, °)

**Table:**

*D*—H···*A*	*D*—H	H···*A*	*D*···*A*	*D*—H···*A*
O3—H3···O2^i^	0.82	1.84	2.659 (5)	172
C25—H25···O2^ii^	0.93	2.63	3.454 (6)	148

Symmetry codes: (i) *x*, *y*+1, *z*; (ii) *x*+1, *y*, *z*.
